# Kidneys From Elderly Deceased Donors—Is 70 the New 60?

**DOI:** 10.3389/fimmu.2019.02701

**Published:** 2019-11-27

**Authors:** Fabian Echterdiek, Vedat Schwenger, Bernd Döhler, Joerg Latus, Daniel Kitterer, Uwe Heemann, Caner Süsal

**Affiliations:** ^1^Department of Nephrology, Klinikum Stuttgart, Stuttgart, Germany; ^2^Institute of Immunology, Heidelberg University Hospital, Heidelberg, Germany; ^3^Department of Nephrology, Klinikum Rechts der Isar, Technical University Munich, Munich, Germany

**Keywords:** kidney transplantation, marginal donor, expanded criteria donor, elderly donor, death censored graft survival, donor age

## Abstract

There is a growing shortage of kidney donors leading to extended transplant waiting times associated with increased mortality. To expand the donor pool, clinicians nowadays regularly accept organs from elderly donors, including those aged ≥70 years. There is only limited and conflicting data whether kidneys from these elderly donors allow for satisfactory allograft outcome rates. To asses this question, the 5-year death censored graft survival of 116,870 adult first deceased donor kidney allograft recipients that were transplanted at European centers between 1997 and 2016 and reported to the “Collaborative Transplant Study” were analyzed using Kaplan–Meier analysis and country stratified Cox regression. The combinations of the two transplant periods 1997–2006 and 2007–2016 with the donor age categories 18–49, 50–59, 60–69, and ≥70 years were considered. From 1997–2006 to 2007–2016, the median donor age increased from 50 to 55 years and the proportion of kidneys from ≥60-year-old donors rose from 24.1 to 38.8%. At the same time, the proportion of kidneys from ≥70-year-old donors more than doubled (6.7 vs. 15.4%). Between 1997–2006 and 2007–2016, the 5-year graft survival improved in all donor age categories. During 2007–2016, the 5-year death censored graft survival of kidneys from ≥70-year-old donors was comparable to that of kidneys from 60 to 69-year-old donors during 1997–2006. This was true both for younger recipients (18–64 years) and older recipients (≥65 years). Among the younger recipients, 45–64-year-old recipients showed the best death censored graft survival rates for kidneys from old donors. In the country-stratified Cox regression analysis, compared to the reference of grafts from 18 to 49-year-old donors, the hazard ratio for grafts from ≥70-year-old donors during 2007–2016 was 1.92, exactly the same as the hazard ratio for grafts from 60 to 69-year-old donors during 1997–2006. Our analysis indicates that within only one further decade (1997–2006 vs. 2007–2016) the 5-year death censored graft survival of kidneys from ≥70-year old donors improved to the level of kidneys from 60 to 69-year-old donors in the previous decade.

## Introduction

Kidney transplantation is the therapy of choice for patients with end stage renal disease (ESRD) and is associated with improved survival rates also in elderly recipients aged ≥70 years (1, 2). Donation from a living donor provides the best outcome rates; however, in many cases there is no living donor available, leaving patients to wait for an organ from a deceased donor whilst staying on maintenance dialysis. Due to a widespread shortage in donor organs, the waiting time for a deceased donor kidney often amounts to several years (3). At the same time, maintenance hemodialysis is associated with a mortality that is up to 10 times greater than the mortality of the general population, reaching up to 20% per year (4). This dilemma has urged clinicians to increase the donor pool by accepting kidneys from suboptimal donors. First in 2002, these donors were categorized as expanded-criteria donors (ECD) (5). ECD were defined as either aged 60 years or older at time of death or as aged 50–59 years with two of the following three criteria (a) history of hypertension, (b) serum creatinine >1.5 mg/dl, or (c) death by cardiovascular accident.

There is ample evidence that kidneys from ECD have worse survival rates than kidneys from standard criteria donors (SCD) (6). Nonetheless, the proportion of ECD has strongly increased in the last decade, especially in Europe, amounting to almost 50% of all deceased donors in recent years (7, 8). Nowadays, clinicians regularly transplant kidneys from deceased donors aged 65 and older: in 2018, 25% of all deceased donor kidneys transplanted within the Eurotransplant (ET) region came from donors aged ≥65 years (7). In this aging donor population with—by nature—an increased amount of (potentially unknown) comorbidities, donor selection has become even more important. There is limited and partly conflicting data whether kidney transplants from donors aged 70 or older result in satisfactory allograft outcome rates. Some transplant centers have reported encouraging results for kidneys from ≥70-year-old donors with graft survival rates comparable to kidneys from younger donors by using pre-implantation biopsies and proceeding with either single or dual-kidney transplantation or discarding the organs, depending on the biopsy results (9, 10). However, graft survival rates of kidneys transplanted within the European Senior Program (ESP) (comprising—by definition—only donors aged ≥65 years) have been shown to be slightly worse than the graft survival rates in the regular Eurotransplant Kidney Allocation System (ETKAS) (11). One large study in the United States (US) also showed significantly worse outcomes for kidneys from donors aged 70 years and older when compared to donors aged 50–69 (12). However, no study so far has assessed how the graft survival rates of kidneys from donors aged ≥70 years that were transplanted in recent years compare to survival rates of kidneys from coeval as well as younger donors obtained in the past. To evaluate this matter, we analyzed outcome data from the international Collaborative Transplant Study (CTS) by combining transplant period and donor age.

## Materials and Methods

### Study Design

First deceased donor kidney transplants in adult recipients and donors (age ≥18 years) reported to CTS were analyzed (www.ctstransplant.org). Multi-organ transplants (e.g., kidney and pancreas) were excluded. Analysis was limited to data from transplant centers in Europe (209 centers from 23 countries). The combination of the two transplant periods 1997–2006 and 2007–2016 with the following four donor age categories 18–49, 50–59, 60–69, and ≥70 years were considered. Moreover, kidney transplants from the two transplant periods were also stratified according to four recipient age categories: 18–44, 45–54, 55–64, and ≥65 years.

### Statistical Analysis and Outcome

The primary endpoint was 5-year death censored graft survival. Categorical variables were assessed using Fisher's exact test or chi-squared test. For continuous variables, the median with interquartile range (IQR) as well as the mean with standard deviation (SD) are shown. Mann–Whitney–*U*-test was used for statistical analysis of continuous variables. Survival rates were illustrated using the Kaplan–Meier method. Hazard ratios of the influence of the donor age categories with 95% confidence intervals (CI) were calculated with multivariable Cox regression. Analyses were stratified by country to eliminate confounding by different country-based allocation strategies. Other parameters such as donor/recipient comorbidities, cold ischemia time, duration of dialysis, induction therapy, sensitization status, or race were deliberately not considered for Cox regression analysis as the primary goal was to show the real-life changes in 5-year death censored graft survival between the two transplant periods for the different donor age groups. To exclude the influence of age-matched allocation strategies, separate analyses in the subgroups of 18–64 and ≥65-year-old recipients were also performed. The survival rate of the 18–49-year-old donors in the period 1997–2006 served as reference.

Two tailed *P*-values of < 0.05 were considered statistically significant. Statistical analysis was conducted using the software IBM^®^ SPSS^®^ Statistics version 25.0 (SPSS Inc., IBM Corporation, Somers, NY, USA).

## Results

In total, 116,870 patients were assessed, 59,158 in the transplant period 1997–2006 and 57,712 patients in the transplant period 2007–2016.

**Figure 1:**
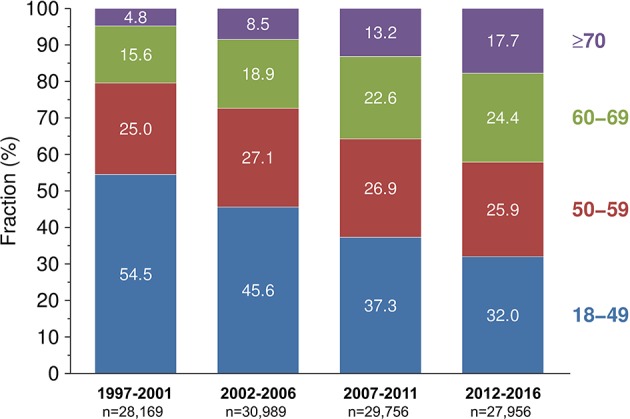
Development of donor age in European adult recipients of first deceased donor kidney transplants across different time periods.

The demographics of study patients from both periods are summarized in Table 1. The median donor age increased from 50 years during 1997–2006 to 55 years during 2007–2016 (*P* < 0.001). Within the donor population, the proportion of 60–69-year-old donors increased significantly from 17.3% during 1997–2006 to 23.4% during 2007–2016 (*P* < 0.001). The absolute number of donors aged ≥70-years more than doubled (3,996 during 1997–2006 vs. 8,874 during 2007–2016) and their relative proportion rose from 6.7 to 15.4% (*P* < 0.001). The median recipient age also increased over time (51 vs. 56 years; *P* < 0.001). Figure 1 visualizes the development of donor age in 5-year intervals over the course of the 20 years assessed: From 1997–2001 to 2012–2016, the proportion of ≥70- as well as 60–69-year-old donors increased from 4.8 and 15.6% to 17.7 and 24.4%, respectively. This was paralleled by a decline of 18–49-year-old donors from 54.5 to 32.0% (*P* < 0.001).

**Table 1:** Demographics of study patients.

**Characteristic**	**Unknown (%)**	**Transplant period**		***P***
		**1997–2006 *n* = 59,158**	**2007–2016 *n* = 57,712**	
Recipient sex	0.0			<0.001
Female		22,185 (37.5)	20,806 (36.1)	
Male		36,964 (62.5)	36,894 (63.9)	
Recipient age (years)	–			<0.001
Median [IQR]		51 [40–60]	56 [46–64]	
Mean ± SD		49.6 ± 12.8	53.9 ± 12.8	
18–64		51,387 (86.9)	44,128 (76.5)	<0.001
≥65		7,771 (13.1)	13,584 (24.5)	
Donor age (years)	–			<0.001
Median [IQR]		50 [38–59]	55 [45–65]	
Mean ± SD		48.2 ± 14.9	54.0 ± 15.0	
18–49		29,477 (49.8)	20,050 (34.7)	<0.001
50–59		15,441 (26.1)	15,255 (26.4)	
60–69		10,244 (17.3)	13,533 (23.4)	
≥70		3,996 (6.7)	8,874 (15.4)	
Cause of donor death	5.8			<0.001
Trauma		15,445 (27.9)	9,549 (17.4)	
Cerebrovascular		34,179 (61.8)	35,009 (63.8)	
Other		5,658 (10.2)	10,276 (18.7)	
Donation after cardiac death	3.2	2,218 (3.9)	7,395 (13.2)	<0.001
Donor history of hypertension	3.4	7,194 (12.5)	8,598 (15.4)	<0.001
Cold ischemia time (hours)	7.9			<0.001
Median [IQR]		17 [13–21]	14 [11–18]	
Mean ± SD		17.7 ± 7.0	14.8 ± 5.6	
HLA-A+B+DR mismatches	10.9			<0.001
Mean ± SD		2.9 ± 1.4	3.3 ± 1.4	
0–1		8,199 (15.1)	5,139 (10.3)	<0.001
2–4		39,330 (72.3)	34,605 (69.5)	
5–6		6,816 (12.5)	10,051 (20.2)	

*IQR, interquartile range; SD, standard deviation; numbers in brackets represent percentages if not otherwise indicated*.

Furthermore, the total number and especially the number of 5–6 HLA-mismatched transplants increased significantly between 1997–2006 and 2007–2016 (2.9 vs. 3.3 and 12.5 vs. 20.2%, respectively; *P* < 0.001 for both comparisons). The donors had significantly more often a history of hypertension (12.5 vs. 15.4%; *P* < 0.001), the cause of donor death was significantly less often trauma (27.9 vs. 17.4%; *P* < 0.001), and donation after cardiac death became more frequent (3.9 vs. 13.2%; *P* < 0.001). Cold ischemia time was the only parameter which improved, i.e., it decreased in median from 17 to 14 h (*P* < 0.001).

**Figure 2:**
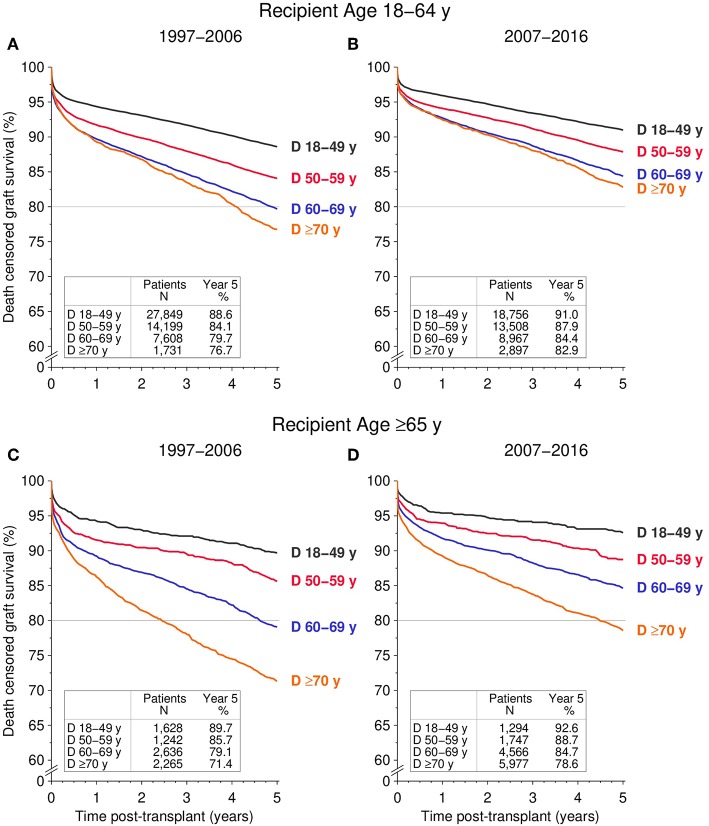
Influence of donor age (D) on death censored graft survival during the first 5 post-transplant years, stratified by recipient age and transplant period. **(A,B)** Display 5-year death censored graft survival for recipients aged 18–64 transplanted during the **(A)** 1997–2006 and **(B)** 2007–2016 period; **(C,D)** display 5-year death censored graft survival for ≥65-year-old recipients for **(C)** 1997–2006 and **(D)** 2007–2016 (all global log rank *P* < 0.001).

Although a negative trend was evident in the majority of the demographic parameters, the 5-year death censored graft survival improved significantly across all donor age groups from 1997–2006 to 2007–2016, including younger recipients aged 18–64-years as well as older recipients aged ≥65-years (Figure 2). In detail: in 18–64-year-old recipients, the 5-year death censored graft survival of kidneys from ≥70-year-old donors during 2007–2016 was superior compared to kidneys from 60 to 69-year-old donors during 1997–2006 (82.9% [95% CI 81.2–84.4%] vs. 79.7% [95% CI 78.7–80.6%], log rank *P* < 0.001, Figures 2A,B). The 5-year death censored graft survival of kidneys from 60 to 69-year-old donors in 2007–2016 improved to the level of kidneys from 50 to 59-year-old donors in 1997–2006 (84.4% [95% CI 83.5–85.2%] vs. 84.1% [95% CI 83.4–84.7%], *P* = 0.27).

**Figure 3:**
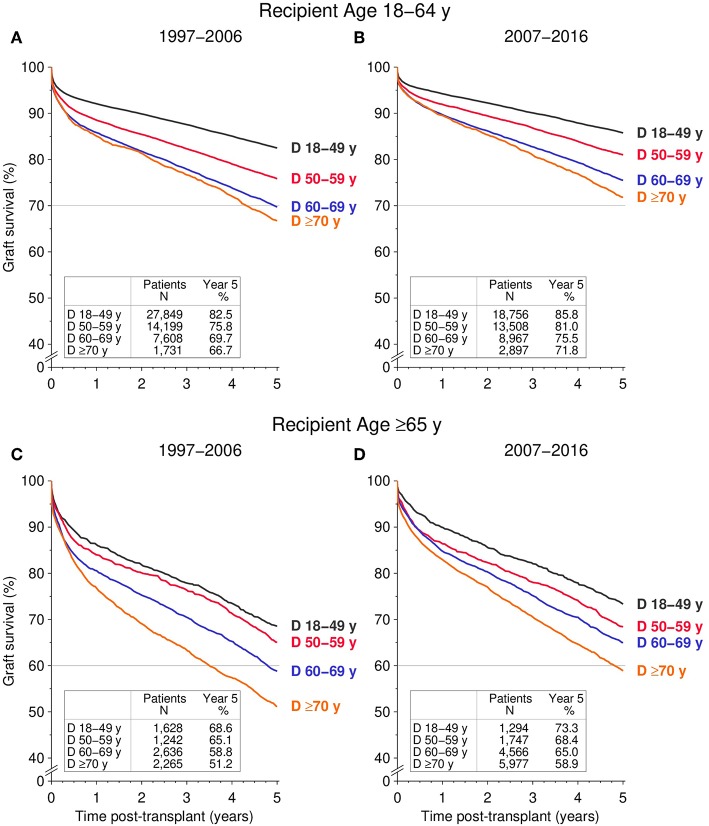
Influence of donor age (D) on all cause graft survival during the first 5 post-transplant years, stratified by recipient age and transplant period. **(A,B)** Display 5-year graft survival for recipients aged 18–64 transplanted during the **(A)** 1997–2006 and **(B)** 2007–2016 period; **(C,D)** display 5-year graft survival for ≥65-year-old recipients for **(C)** 1997–2006 and **(D)** 2007–2016 (all global log rank *P* < 0.001).

In ≥65-year-old recipients, the 5-year death censored graft survival of kidneys from ≥70-year-old donors during 2007–2016 was similar to kidneys from 60 to 69-year-old donors transplanted during 1997–2006 (78.6% [95% CI 77.3–79.8%] vs. 79.1% [95% CI 77.3–80.7%], *P* = 0.60, Figures 2C,D). Likewise, the 5-year death censored graft survival of kidneys from 60 to 69-year-old donors transplanted during 2007–2016 was comparable to kidneys from 50 to 59-year old donors during 1997–2006 (84.7% [95% CI 83.4%−85.9%] vs. 85.7% [95% CI 83.4%−87.6%], *P* = 0.45). The same results were obtained when comparing all cause graft survival among the different donor age groups across the two transplant periods—both in young recipients (18–64 years) and old recipients (≥65 years; Figure 3).

**Figure 4:**
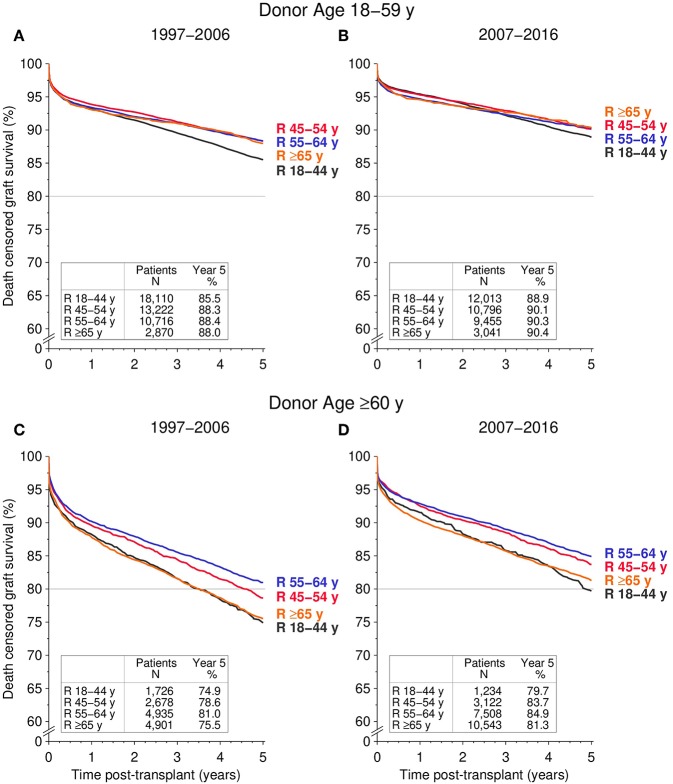
Influence of recipient age (R) on death censored graft survival during the first 5 post-transplant years, stratified by donor age and transplant period. **(A,B)** Display 5-year death censored graft survival for donors aged 18–59 transplanted during the **(A)** 1997–2006 and **(B)** 2007–2016 period; **(C,D)** display 5-year death censored graft survival for ≥60-year-old donors for **(C)** 1997–2006 and **(D)** 2007–2016 [global log rank **(B)** *P* = 0.12; **(A,C,D)** *P* < 0.001].

The influence of recipient age on 5-year death censored graft survival was also assessed for the two different transplant periods (Figure 4). Except for 18–44-year-old recipients, kidneys from young donors (aged 18–59 years) showed similar survival rates in all recipient age groups of the two considered transplant periods (global log rank *P* = 0.87 and *P* = 0.82, respectively). Kidneys from older donors (aged ≥60 years) had significantly worse 5-year death censored graft survival rates in 18–44-year-old as well as in ≥65-year-old recipients compared to 45–64-year-old recipients, regardless of transplant period (all log rank *P* < 0.001).

In the Cox regression analysis of death censored graft loss stratified by country, the 5-year graft loss of kidneys from 18 to 49-year-old donors transplanted in the 1997–2006 period was taken as reference (Table 2). When all recipients were analyzed together, the hazard ratio for graft loss of kidneys from ≥70-year-old donors during 2007–2016 was 1.92 (95% CI 1.80–2.05), the same as the hazard ratio for kidneys from 60 to 69-year-old donors during 1997–2006 (95% CI 1.81–2.03; *P* = 0.96). In addition, the hazard ratio for kidneys from 60 to 69-year-old donors during 2007–2016 was the same as the hazard ratio for kidneys from 50 to 59-year-old donors during 1997–2006 (1.45, 95% CI 1.37–1.54 and 1.38–1.53, respectively; *P* = 0.96).

**Table 2:** Results of the Cox regression analysis for influence of donor age on death censored graft loss during the first 5 post-transplant years.

**Transplant period and donor age**		***N***	**HR**	**95% CI**	***P-*value**
**All recipients**					
1997–2006	18–49 years	29,477	1 (ref)	–	–
	50–59 years	15,441	1.45	1.38–1.53	<0.001
	60–69 years	10,244	1.92	1.81–2.03	<0.001
	≥70 years	3,996	2.53	2.35–2.73	<0.001
2007–2016	18–49 years	20,050	0.80	0.75–0.85	<0.001
	50–59 years	15,255	1.10	1.03–1.17	0.003
	60–69 years	13,533	1.45	1.37–1.54	<0.001
	≥70 years	8,874	1.92	1.80–2.05	<0.001
**Recipients 18–64 years**					
1997–2006	18–49 years	27,849	1 (ref)	–	–
	50–59 years	14,199	1.45	1.37–1.54	<0.001
	60–69 years	7,608	1.93	1.81–2.06	<0.001
	≥70 years	1,731	2.38	2.13–2.65	<0.001
2007–2016	18–49 years	18,756	0.80	0.75–0.85	<0.001
	50–59 years	13,508	1.10	1.03–1.17	0.006
	60–69 years	8,967	1.46	1.37–1.57	<0.001
	≥70 years	2,897	1.68	1.51–1.87	<0.001
**Recipients** **≥65 years**					
1997–2006	18–49 years	1,628	1 (ref)	–	–
	50–59 years	1,242	1.52	1.21–1.90	<0.001
	60–69 years	2,636	1.96	1.63–2.36	<0.001
	≥70 years	2,265	2.70	2.24–3.25	<0.001
2007–2016	18–49 years	1,294	0.77	0.59–1.02	0.064
	50–59 years	1,747	1.23	0.99–1.55	0.066
	60–69 years	4,566	1.53	1.27–1.83	<0.001
	≥70 years	5,977	2.15	1.80–2.57	<0.001

*Hazard ratios (HR) with 95% confidence interval (CI) of donor age are shown*.

In 18–64-year-old recipients, the hazard ratio for graft loss of kidneys from ≥70-year-old donors during 2007–2016 was lower (1.68, 95% CI 1.51–1.87) compared to that of kidneys from ≥70-year- as well as 60–69-year-old donors during 1997–2006 (2.38; 95% CI 2.13–2.65; *P* < 0.001 and 1.93; 95% CI 1.81–2.06; *P* = 0.017, respectively) and slightly (but significantly) worse than the hazard ratio for kidneys from 50 to 59-year-old donors during 1997–2006 (1.45; 95% CI 1.37–1.54; *P* = 0.008). The hazard ratio for kidneys from 60 to 69-year-old donors during 2007–2016 was comparable to that for kidneys from 50 to 59-year-old donors during 1997–2006 (1.46, 95% CI 1.37–1.57 and 1.45; 95% CI 1.37–1.54, respectively; *P* = 0.83).

In ≥65-year-old recipients, the hazard ratio for graft loss of kidneys from ≥70-year-old donors during 2007–2016 was lower compared to ≥70-year-old donors during 1997–2006 (2.15, 95% CI 1.80–2.57 and 2.70, 95% CI 2.24–3.25, respectively; *P* < 0.001) and only slightly (but not significantly) worse compared to 60–69 year-old-donors during 1997–2006 (1.96, 95% CI 1.63–2.36; *P* = 0.097). The hazard ratio of kidneys from 60 to 69-year-old donors during 2007–2016 and that of kidneys from 50 to 59-year-old donors during 1997–2006 were nearly equal (1.53, 95% CI 1.27–1.83 and 1.52; 95% CI 1.21–1.90, respectively; *P* = 0.95).

## Discussion

We could demonstrate that within only one decade the 5-year death censored graft survival rates of kidneys from donors aged ≥70 years improved to a level that was comparable to the graft survival of kidneys from donors aged 60–69 years in the previous decade. Moreover, as may have been expected—a significant 5-year increase in median donor age was observed during the same time period. Remarkably, the proportion of donors aged ≥70 years more than doubled from 6.7 to 15.4%. Regardless of the changes in donor age distribution, graft survival improved significantly in all donor age groups over the assessed time period.

Increasing donor age is widely recognized as one of the most important risk factors for poor kidney allograft survival (13–15). As a consequence, the discard rate of kidneys from elderly donors is strongly elevated, especially in donors aged ≥65 years (10, 16–18). Nonetheless, we were able to show that the graft survival of kidneys from donors aged ≥70 years improved, in the short time interval of one decade, to the level previously seen for kidneys from donors aged 60–69 years. At the same time, the graft survival of kidneys from 60 to 69-year-old donors improved to the level of 50–59-year old donors from the previous decade. In times of universal organ shortage, these are remarkable findings especially considering the high organ discard rate in old donors mentioned above. Of course, it has to be pointed out that kidneys from younger donors still perform distinctly better than kidneys from older donors and that increasing donor age remains a negative predictor of graft survival. However, the absolute and relative improvements in 5-year death censored graft survival and all cause graft survival of kidneys from older donors over the course of just one decade are astonishing. This is an important, reassuring finding for clinicians when deciding on whether to accept an organ offer from an elderly donor or not.

Several previous publications had shown fairly poor survival rates for kidney grafts from old donors transplanted into young recipients (11, 12, 19). However, our data show, that the hazard ratio of kidneys from ≥70-year-old donors transplanted into 18–64-year-old recipients decreased—within only one decade—from 2.38 to 1.68, which is better than the hazard ratio reported for 60–69-year-old kidney donors during 1997–2006 (1.93) and only slightly worse than the hazard ratio reported for 50–59-year-old kidney donors (1.45). It needs to be pointed out though that not all young recipients fare alike with kidneys of older donors. We demonstrate that it is the group of 45–64-year-old recipients that show the best 5-year death censored graft survival rates whereas 18–44-year-old recipients have a significantly reduced graft survival. Therefore, if kidneys from older donors (age ≥60 years) are transplanted into younger recipients (<65 years), they should be chosen primarily for the group of 45–64-year-old recipients. Moreover, out data indicate that a strict old-for-old allocation concept puts 45–64-year-old recipients at a disadvantage as they also profit from a ≥60-year-old-donor.

We can only speculate about the main factors that are responsible for the improved graft survival rates: post-transplant surveillance has improved, ranging from more frequent, in some centers to even per-protocol kidney biopsies with more standardized histological evaluation; close surveillance of individualized immunosuppressive drug levels, regular screening for development of donor-specific antibodies, effective antiviral prophylaxis and better diagnosis and treatment of concomitant, cardiovascular and renal risk factors. Furthermore, there have been advances in the pre- and peri-transplant period including more sensitive alloantibody detection, revised allocation procedures and improved kidney storage and preservation. The impact of different immunosuppressive agents on graft survival is controversial with some studies suggesting superior outcomes with tacrolimus and mycophenolate but other large studies showing no difference (20–24). What seems more important is to tailor the choice of immunosuppressive agents to the immunological risk profile of each patient as well as to consider individual patient risk factors such as co-morbidities and the clinical course after transplant, especially in elder recipients (25). All factors mentioned above might—to varying degrees—have contributed to the improved survival rates in transplants from elderly donors (26–28).

The general increase in 5-year death censored graft survival is even more noteworthy considering the increased immunological risk that clinicians were willing to take in the more recent transplant period. The mean number of HLA-mismatches increased significantly, from 2.9 during 1997–2006 to 3.3 during 2007–2016. Furthermore, the number of kidney transplants with 5 or 6 HLA-mismatches increased from 12.5 to 20.2%. It has been well-documented that the number of HLA-mismatches is strongly associated with worse long-term graft survival (29, 30). Apparently, the aforementioned improvements both in the peri- and post-transplant management seem to have outweighed the enhanced immunological risk.

There have been previous studies on kidney transplantations from elderly donors aged ≥70 years. Several Italian and British studies have shown that performing pre-implantation biopsies of donor kidneys aged ≥70 years and then proceeding with either dual or single transplantation or discarding the organs depending on the histological evaluation resulted in kidney graft survival rates that were equal to survival rates of organs from younger donors (9, 10, 31). In contrast, studies on the European Senior Programme have reported slightly worse survival rates for kidneys from donors aged ≥65 years when compared to the regular ETKAS programme (11). There is also a large study from the United States that presented inferior survival data for kidneys from donors aged ≥70 years (12). Of note, in all these studies the survival of kidneys from older donors was compared to that of younger donors from the same time period. Our data are novel as we compared the (death censored) graft survival of different kidney donor age groups to the same age cohorts transplanted 10 years earlier. This allowed us to appreciate the significant improvements, especially for elderly donor kidneys, that have been achieved over the last 20 years. Our data also stress that this improvement was necessary for we have also seen a remarkable change in kidney donor characteristics in Europe. The median donor age increased to 55 years and 17.7% of donors were ≥70 years old during 2012–2016. At the same time, the proportion of 18–49-year-old donors decreased from 55% in 1997–2001 to 32% in 2012–2016. Nowadays, ECD seem to have almost become the new average donor, at least in Europe. Interestingly, these trends are not observed in the US where kidneys from ≥65-year-old donors still comprise <5% of the donor pool with no upward trend during the last decade (18). In contrast, our data from European centers demonstrate that even kidney allografts from donors aged ≥70 years can be accepted with good outcome rates for selected recipients.

Our study has several limitations. First, we cannot fully exclude a potential center selection bias as the data in our dataset were not collected at random but rather stemmed from the participants of the CTS, a voluntary network of transplant centers worldwide. However, the data of this study originated from more than 200 centers in 23 European countries, comprising a total of 116,870 patients. About two thirds of the data set came from countries, where all (or nearly all) of the countries' transplant centers report their data to CTS. Moreover, the CTS has excellent follow-up completeness rates of 97% 1 year and 95% 5 years post-transplant (32). Therefore, we consider the impact of a potential center selection bias to be marginal. Second, our study focussed on donor age. We deliberately did not consider other parameters that are known to be associated with graft survival such as donor/recipient comorbidities (arterial hypertension/diabetes mellitus), duration of dialysis, cold ischemia time, or number of HLA-mismatches in the Cox regression analysis. This was done as we wanted to illustrate the real-life improvements in graft survival for kidneys from old donors that have been achieved within just one decade irrespective of potential changes in other variables. However, looking at the parameters that were available within the CTS, except for cold ischemia time, all factors with a negative impact on graft survival were more frequent in the more recent transplant period (number of HLA-mismatches, donor history of arterial hypertension, donation after cardiac death, cerebrovascular accident as cause of death, high recipient age). Cold ischemia time was the only measured parameter that was in favor of the second transplant period as it was found to have diminished from 1997–2006 to 2007–2016. We are aware that this brief overview is by no means equivalent to a full regression analysis correcting for potential confounders; however, from the data available we have no evidence that the pattern of our results could be due to a strong bias. Third, we focused our analysis primarily on death censored graft survival and did not report on patient survival. We chose to do so because the life expectancy of kidney transplant patients increased over the course of the 20-year time period assessed in the paper (33). However, this fact itself already improves patient survival thus impairing a correct analysis of this parameter. Death censored graft survival purely reflects graft function independent of patient survival data which is why we chose to focus on it. Importantly, the all cause graft survival data reported by us confirm the findings from the analysis of death censored graft survival. Forth, we do not report follow-up data beyond 5-years post-transplant, because the long-term data available for the second transplant period (2007–2016) is still rather incomplete after year 5. Hence, we do not know if the findings demonstrated in this study will persist long-term. However, the hazard ratio of (death censored) graft survival usually remains approximately constant after the first-year post-transplant, suggesting that the long-term effects will be similar to our findings 5-years after transplantation.

In conclusion, we demonstrate that within only one decade, namely from 1997–2006 to 2007–2016, the 5-year death censored graft survival of kidneys from ≥70-year-old donors improved to a level of kidneys from 60 to 69-year-old donors in the previous decade. The same improvement was observed also for kidneys from 60 to 69-year-old donors compared to kidneys from 50 to 59-year-old donors transplanted one decade earlier. Considering the unmet lack of donor organs, these results may help to further expand the kidney donor pool especially for recipients aged ≥45 years.

## Data Availability Statement

The raw data are available upon request to the Collaborative Transplant Study in accordance with the consents of the patients, the participating transplant centers and registries.

## Ethics Statement

The Collaborative Transplant Study involving human participants was reviewed and approved by the ethics committee of the Medical Faculty of Heidelberg University (No. 083/2005) and performed in accordance with the World Medical Association Declaration of Helsinki Ethical Principles in the currently valid version. The patients/participants provided their written informed consent to participate in this study.

## Author Contributions

FE, VS, BD, and CS drafted and wrote the manuscript. FE, VS, JL, DK, UH, and CS conceived the idea of the manuscript. BD and CS provided the CTS database and performed statistical analysis. All authors reviewed and edited the manuscript and provided final approval for publication.

### Conflict of Interest

The authors declare that the research was conducted in the absence of any commercial or financial relationships that could be construed as a potential conflict of interest.

## References

[1] Rao PS Merion RM Ashby VB Port FK Wolfe RA Kayler LK. Renal transplantation in elderly patients older than 70 years of age: results from the scientific registry of transplant recipients. Transplantation. (2007) 83:1069–74. 10.1097/01.tp.0000259621.56861.31 17452897

[2] Heldal K Hartmann A Grootendorst DC de Jager DJ Leivestad T Foss A. Benefit of kidney transplantation beyond 70 years of age. Nephrol Dial Transplant. (2010) 25:1680–7. 10.1093/ndt/gfp681 20038521 PMC2856560

[3] Wu DA Watson CJ Bradley JA Johnson RJ Forsythe JL Oniscu GC. Global trends and challenges in deceased donor kidney allocation. Kidney Int. (2017) 91:1287–99. 10.1016/j.kint.2016.09.054 28320531

[4] Collins AJ Foley RN Chavers B Gilbertson D Herzog C Ishani A. US renal data system 2013 annual data report. Am J Kidney Dis. (2014) 63:A7. 10.1053/j.ajkd.2013.11.001 24360288

[5] Metzger RA Delmonico FL Feng S Port Fk Wynn JJ Merion R. Expanded criteria donors for kidney transplantation. Am J Transplant. (2003) 3(Suppl. 4):114–25. 10.1034/j.1600-6143.3.s4.11.x 12694055

[6] Querard AH Foucher Y Combescure C Dantan E Larmet D Lorent M. Comparison of survival outcomes between expanded criteria donor and standard criteria donor kidney transplant recipients: a systematic review and meta-analysis. Transpl Int. (2016) 29:403–15. 10.1111/tri.12736 26756928

[7] ET Eurotransplant Statistics Report Library. Available online at: http://statistics.eurotransplant.org/

[8] Coemans M Süsal C Döhler B Anglicheau D Giral M Bestard O. Analyses of the short- and long-term graft survival after kidney transplantation in Europe between 1986 and 2015. Kidney Int. (2018) 94:964–73. 10.1016/j.kint.2018.05.018 30049474

[9] Ruggenenti P Silvestre C Boschiero L Rota G Furian L Perna A. Long-term outcome of renal transplantation from octogenarian donors: a multicenter controlled study. Am J Transplant. (2017) 17:3159–71. 10.1111/ajt.14459 28792681

[10] Messina M Diena D Dellepiane S Guzzo G Lo Sardo L Fop F. Long-term outcomes and discard rate of kidneys by decade of extended criteria donor age. Clin J Am Soc Nephrol. (2017) 12:323–31. 10.2215/CJN.06550616 27979977 PMC5293338

[11] Frei U Noeldeke J Machold-Fabrizii V Arbogast H Margreiter R Fricke L. Prospective age-matching in elderly kidney transplant recipients–a 5-year analysis of the Eurotransplant Senior Program. Am J Transplant. (2008) 8:50–7. 10.1111/j.1600-6143.2007.02014.x 17973969

[12] Chavalitdhamrong D Gill J Takemoto S Madhira BR Cho YW Shah T. Patient and graft outcomes from deceased kidney donors age 70 years and older: an analysis of the organ procurement transplant network/united network of organ sharing database. Transplantation. (2008) 85:1573–9. 10.1097/TP.0b013e31817059a1 18551062

[13] Rao PS Schaubel DE Guidinger MK Andreoni KA Wolfe RA Merion RM. A comprehensive risk quantification score for deceased donor kidneys: the kidney donor risk index. Transplantation. (2009) 88:231–6. 10.1097/TP.0b013e3181ac620b 19623019

[14] Veroux M Grosso G Corona D Mistretta A Giaquinta A Giuffrida G. Age is an important predictor of kidney transplantation outcome. Nephrol Dial Transplant. (2012) 27:1663–71. 10.1093/ndt/gfr524 21926404

[15] Dayoub JC Cortese F AnŽič A Grum T de Magalhães JP. The effects of donor age on organ transplants: a review and implications for aging research. Exp Gerontol. (2018) 110:230–40. 10.1016/j.exger.2018.06.019 29935294 PMC6123500

[16] Hall IE Schröppel B Doshi MD Ficek J Weng FL Hasz RD. Associations of deceased donor kidney injury with kidney discard and function after transplantation. Am J Transplant. (2015) 15:1623–31. 10.1111/ajt.13144 25762442 PMC4563988

[17] Massie AB Desai NM Montgomery RA Singer AL Segev DL Improving distribution efficiency of hard-to-place deceased donor kidneys: predicting probability of discard or delay. Am J Transplant. (2010) 10:1613–20. 10.1111/j.1600-6143.2010.03163.x 20642686

[18] Hart A Smith JM Skeans MA Gustafson SK Wilk AR Castro S. OPTN/SRTR 2017 annual data report: kidney. Am J Transplant. (2019) 19(Suppl. 2):19–123. 10.1111/ajt.15274 30811893

[19] Pascual J Zamora J Pirsch JD. A systematic review of kidney transplantation from expanded criteria donors. Am J Kidney Dis. (2008) 52:553–86. 10.1053/j.ajkd.2008.06.005 18725015

[20] Opelz G Dohler B Collaborative Transplant S. Influence of immunosuppressive regimens on graft survival and secondary outcomes after kidney transplantation. Transplantation. (2009) 87:795–802. 10.1097/TP.0b013e318199c1c7 19300179

[21] Pascual M Theruvath T Kawai T Tolkoff-Rubin N Cosimi AB. Strategies to improve long-term outcomes after renal transplantation. N Engl J Med. (2002) 346:580–90. 10.1056/NEJMra011295 11856798

[22] Krämer BK Montagnino G Krüger B Margreiter R Olbricht CJ Marcen R. Efficacy and safety of tacrolimus compared with ciclosporin-A in renal transplantation: 7-year observational results. Transpl Int. (2016) 29:307–14. 10.1111/tri.12716 26565071

[23] Wang K Zhang H Li Y Wei Q Li H Yang Y. Efficacy of mycophenolate mofetil versus azathioprine after renal transplantation: a systematic review. Transplant Proc. (2004) 36:2071–2. 10.1016/j.transproceed.2004.07.059 15518749

[24] Webster AC Woodroffe RC Taylor RS Chapman JR Craig JC. Tacrolimus versus ciclosporin as primary immunosuppression for kidney transplant recipients: meta-analysis and meta-regression of randomised trial data. BMJ. (2005) 331:810. 10.1136/bmj.38569.471007.AE 16157605 PMC1246079

[25] Singh P Ng YH Unruh M. Kidney transplantation among the elderly: challenges and opportunities to improve outcomes. Adv Chronic Kidney Dis. (2016) 23:44–50. 10.1053/j.ackd.2015.11.002 26709062

[26] Wekerle T Segev D Lechler R Oberbauer R. Strategies for long-term preservation of kidney graft function. Lancet. (2017) 389:2152–62. 10.1016/S0140-6736(17)31283-7 28561006

[27] Neuberger JM Bechstein WO Kuypers DR Burra P Citterio F De Geest S. Practical recommendations for long-term management of modifiable risks in kidney and liver transplant recipients: a guidance report and clinical checklist by the consensus on managing modifiable risk in transplantation (COMMIT) group. Transplantation. (2017) 101:S1–56. 10.1097/TP.0000000000001651 28328734

[28] Nankivell BJ Kuypers DR. Diagnosis and prevention of chronic kidney allograft loss. Lancet. (2011) 378:1428–37. 10.1016/S0140-6736(11)60699-5 22000139

[29] Opelz G Döhler B. Effect of human leukocyte antigen compatibility on kidney graft survival: comparative analysis of two decades. Transplantation. (2007) 84:137–43. 10.1097/01.tp.0000269725.74189.b9 17667803

[30] Williams RC Opelz G McGarvey CJ Weil EJ Chakkera HA. The risk of transplant failure with HLA mismatch in first adult kidney allografts from deceased donors. Transplantation. (2016) 100:1094–102. 10.1097/TP.0000000000001115 26901078 PMC8086563

[31] Mallon DH Riddiough GE Summers DM Butler AJ Callaghan CJ Bradbury LL Successful transplantation of kidneys from elderly circulatory death donors by using microscopic and macroscopic characteristics to guide single or dual implantation. Am J Transplant. (2015) 15:2931–9. 10.1111/ajt.13349 26108421

[32] Opelz G Döhler B Ruhenstroth A Cinca S Unterrainer C Stricker L. The collaborative transplant study registry. Transplant Rev. (2013) 27:43–5. 10.1016/j.trre.2013.01.004 23465693

[33] Wolfe RA Roys EC Merion RM. Trends in organ donation and transplantation in the United States, 1999–2008. Am J Transplant. (2010) 10:961–72. 10.1111/j.1600-6143.2010.03021.x 20420646

